# Supplementary material for: Mechanism of Antifungal Action of Monoterpene Isoespintanol against Clinical Isolates of Candida tropicalis
Source: Molecules. 2022 Sep 8;27(18):5808. doi: 10.3390/molecules27185808 (PMC9505055; doi:10.3390/molecules27185808)
Supplement: Supplementary file 1 [file molecules-27-05808-s001.zip › molecules-1896329-supplementary.pdf]

Supplementary Materials

Table S1. *Extraction of genomic DNA of Candida tropicalis*

Fungal isolation	*Quantification to 260 nm [ng/µL]*	*Relationship 260/280*
Candida tropicalis	33	1.94

Table S2. *Candida tropicalis NGS sequencing results*

Isolation	*Total read bases (bp)*	*Total reads*	*GC(%)*	*Q30(%)*	*% Clean reads*
C. tropicalis	*2,293,112,878*	*15,186,178*	*33.99*	*90.38*	*96.5*

Table S3. *Statistical results of the assembly of Candida tropicalis.*

Parameter	*Values*
Total length of sequence (bp)	16432470 bp
Total number of sequences	8958
Average contig length	1834 bp
Largest contig (pb)	993367 bp
N50 stats	31413 bp
GC %	32.86

Table S4. *% Growth reduction of Candida tropicalis isolates exposed to ISO (MIC of each isolate).*

ISO	CLI 001	CLI 002	CLI 003	CLI 004	CLI 005	CLI 006	CLI 007
**31.25**	0.5 ± 0.6	8.7 ± 0.7	14.15 ± 1.9	10.52 ± 3.2	1.1 ± 0.7	11.6 ± 9.0	10.1 ± 3.8
**62.5**	2.7 ± 3.7	16.6 ± 0.6	27.21 ± 1.6	30.43 ± 2.1	11.6 ± 3.4	17.9 ± 1.2	16.8 ± 1.0
**125**	6.2 ± 2.9	86.5 ± 2.4	50.91 ± 1.7	55.79 ± 3.3	21.8 ± 4.5	41.5 ± 2.2	56.4 ± 0.7
**250**	81.3 ± 3.5	109.7 ± 0.3	103.81 ± 0.4	101.09 ± 3.0	96 ± 0.3	95.7 ± 0.3	110.9 ± 3.7
**500**	104.4 ± 3.5	157.9 ± 3.0	117.05 ± 0.0	115.47 ± 3.2	103.7 ± 0.9	105.8 ± 0.2	144.5 ± 1.4
**1000**	181.8 ± 9.5	162 ± 2.3	156.12 ± 0.6	152.4 ± 1.3	150.4 ± 1.2	152.8 ± 2.2	147.8 ± 2.4

Table S5. *Viability percentages with MTT*

	Isolations						
**ISO (μg/mL)**	**001**	**002**	**003**	**004**	**005**	**006**	**007**
**31.25**	109.1 ± 9.2	101.8 ± 3.5	102.0 ± 4.7	101.3 ± 10.4	111.7 ± 3.8	107.7 ± 13.4	100.6 ± 17.5
**62.5**	108.6 ± 7.0	65.9 ± 2.5	101.7 ± 1.0	100.6 ± 10.2	107.1 ± 2.7	100.8 ± 12.4	74.0 ± 10.8
**125**	101.3 ± 7.5	56.3 ± 2.1	59.2 ± 2.2	57.4 ± 5.7	104.6 ± 3.6	100.1 ± 12.3	67.0 ± 37.2
**250**	50.0 ± 3.8	8.7 ± 0.6	14.2 ± 0.5	10.2 ± 2.0	48.0 ± 2.1	51.7 ± 5.7	0.0 ± 0.1
**500**	0.9 ± 0.7	1.8 ± 0.2	0.3 ± 0.0	0.0 ± 0.6	1.3 ± 0.1	0.9 ± 0.1	0.0 ± 0.3
**1000**	1.1 ± 0.3	0.0 ± 0.3	0.8 ± 0.1	0.6 ± 0.1	0.0 ± 0.3	0.2 ± 0.1	0.0 ± 0.1
**IC_{50}**	236.8	103.1	141.8	136.4	230.9	235.2	113.7

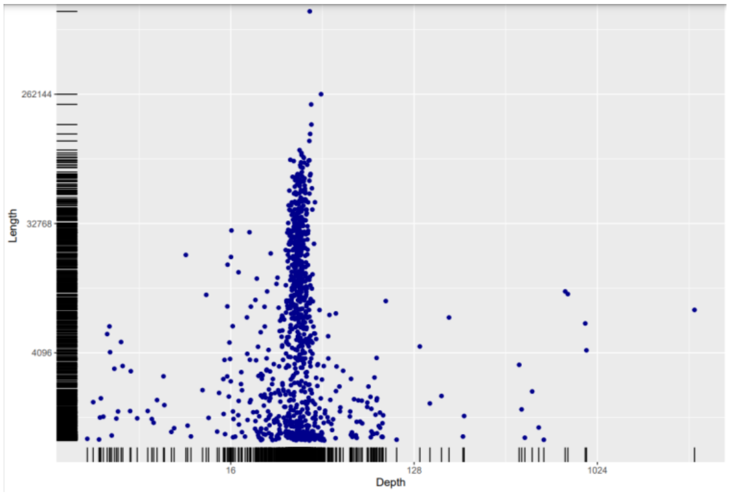

Figure S1. Dot plots created in R, showing the mean depth (DEPTH) and the length of the scaffold (LENGTH) for the *C. tropicalis* isolate analyzed.

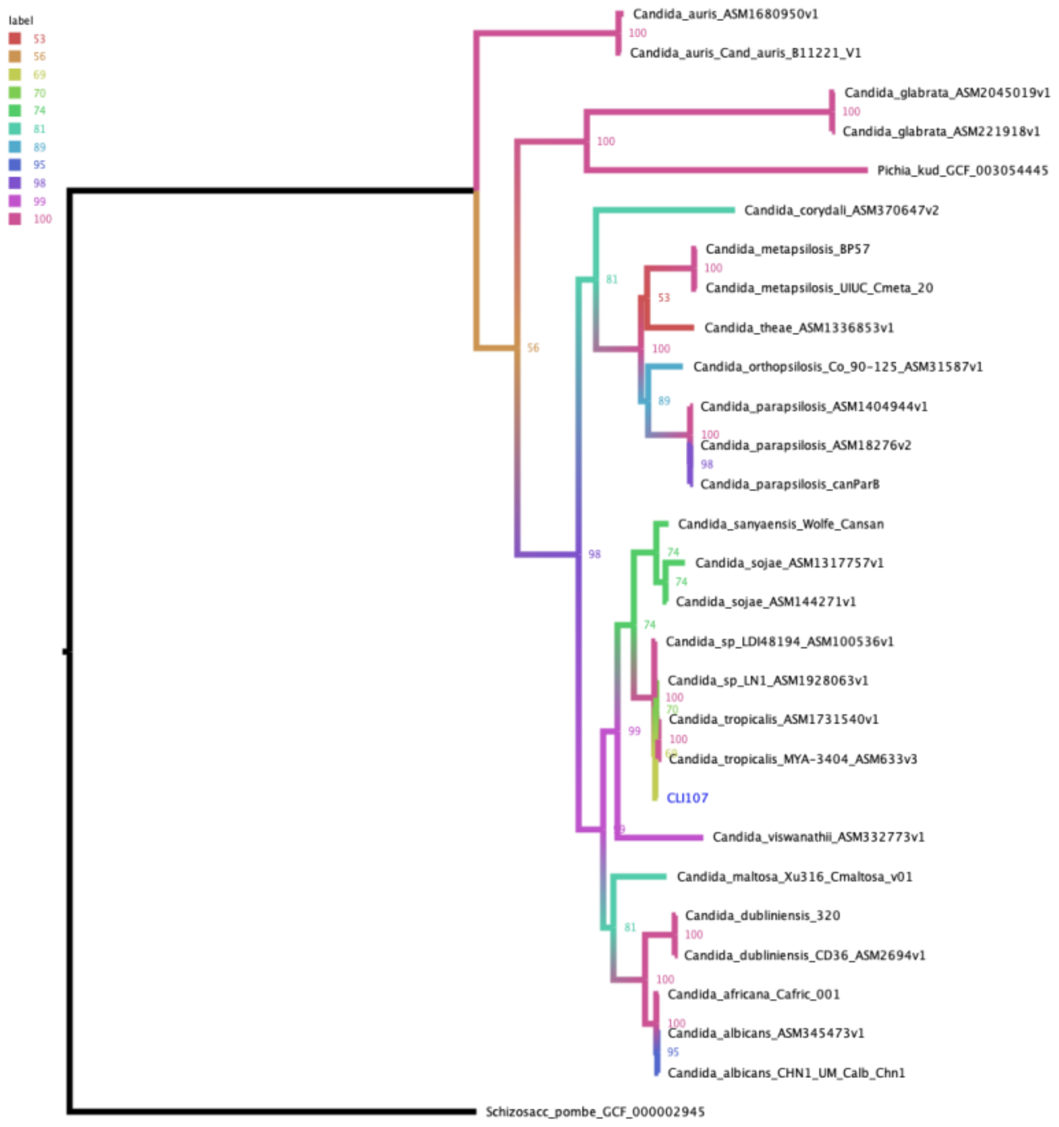

Figure S2. Phylogenetic tree (Maximum Likelihood) based on single-copy conserved genes of *C. tropicalis* strains. CLI107 corresponds to the isolate under study.

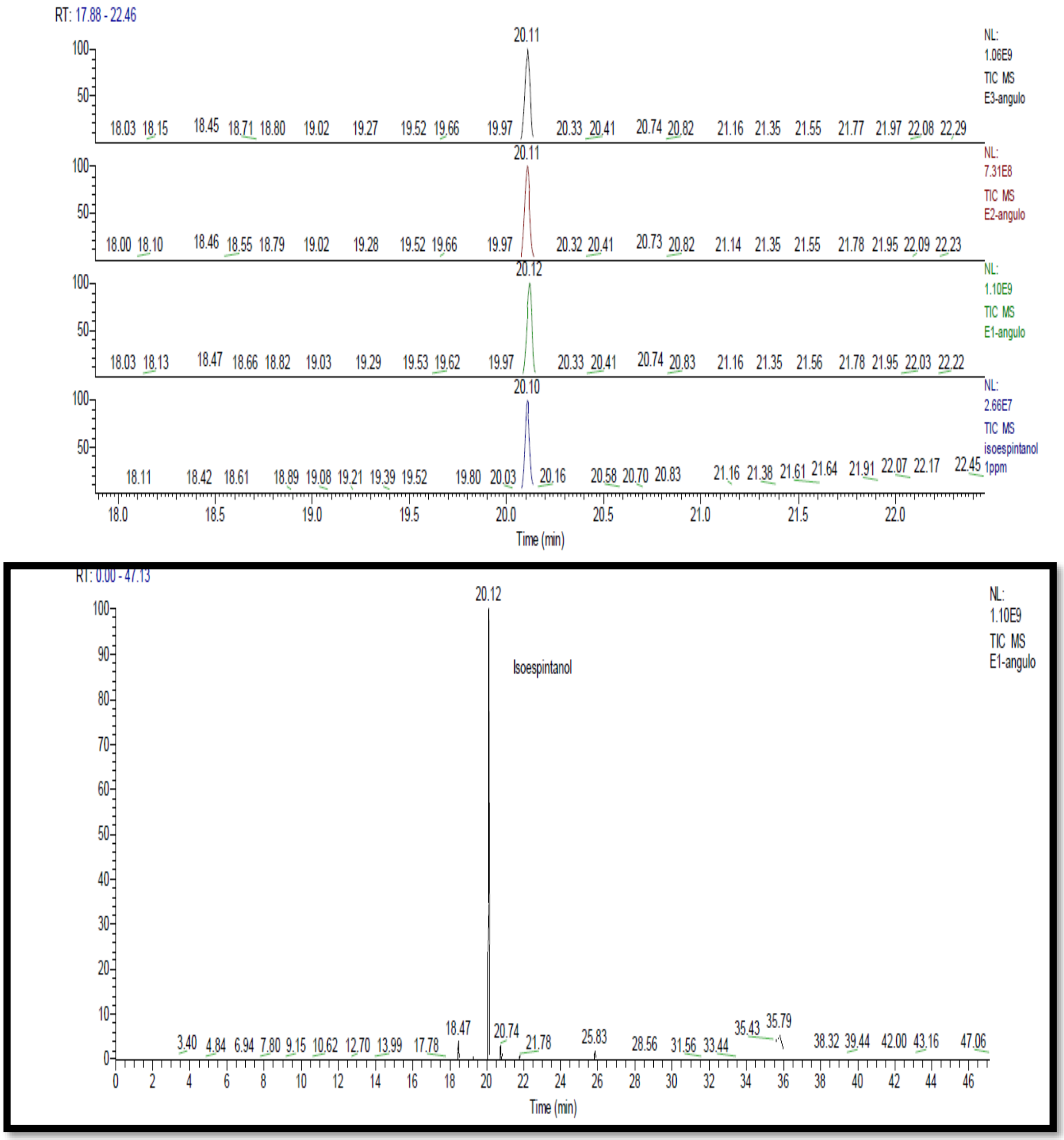

Figure S3. Chromatograms of the purification of Isoespintanol.

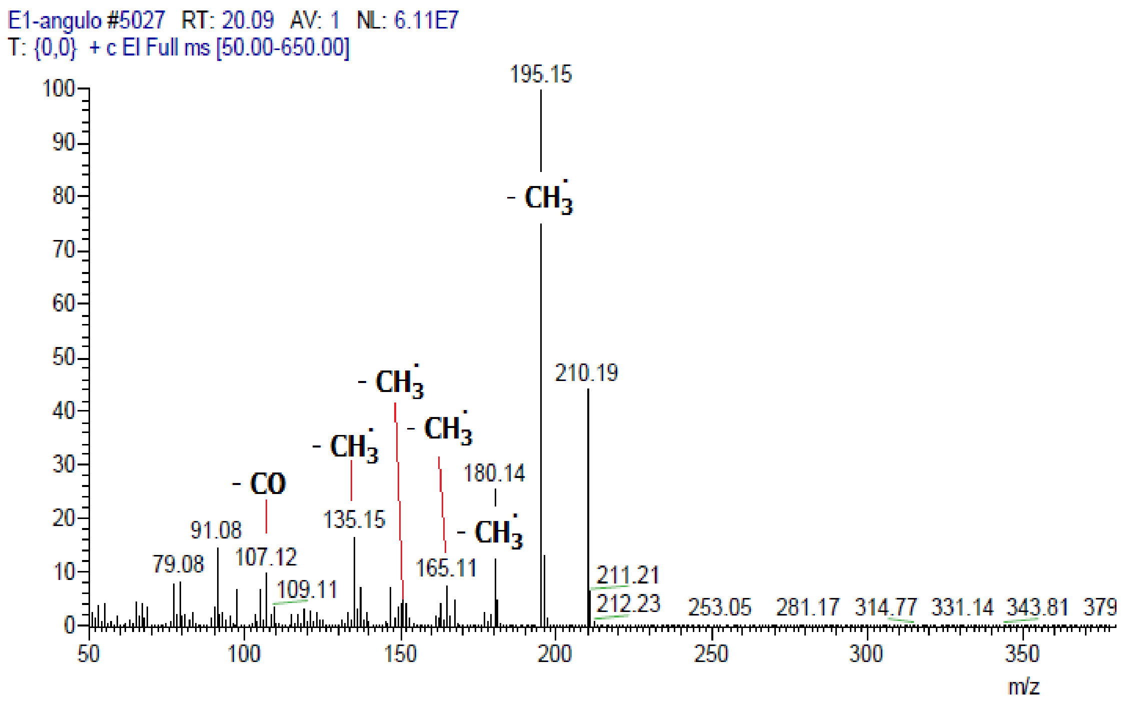

Figure S4. EI-MS spectra of isoespintanol
